# Crystal structure of febuxostat–acetic acid (1/1)

**DOI:** 10.1107/S2056989015005708

**Published:** 2015-04-09

**Authors:** Min Wu, Xiu-Rong Hu, Jian-Ming Gu, Gu-Ping Tang

**Affiliations:** a Chemistry Department, Zhejiang University, Hangzhou, Zhejiang 310028, People’s Republic of China

**Keywords:** crystal structure, febuxostat, acetic acid, co-crystal, hydrogen bonding, π–π stacking

## Abstract

The asymmetric unit of the title compound [systematic name: 2-(3-cyano-4-iso­butyl­oxyphen­yl)-4-methyl­thia­zole-5-carb­oxy­lic acid–acetic acid (1/1)], C_16_H_16_N_2_O_3_S·CH_3_COOH, contains a febuxostat mol­ecule and an acetic acid mol­ecule. In the febuxostat mol­ecule, the thia­zole ring is nearly coplanar with the benzene ring [dihedral angle = 3.24 (2)°]. In the crystal, the febuxostat and acetic acid mol­ecules are linked by O—H⋯O, O—H⋯N hydrogen bonds and weak C—H⋯O hydrogen bonds, forming supra­molecular chains propagating along the *b-*axis direction. π–π stacking is observed between nearly parallel thia­zole and benzene rings of adjacent mol­ecules; the centroid-to-centroid distances are 3.8064 (17) and 3.9296 (17) Å.

## Related literature   

For general apllications of febuxostat in medicine, see: Pascual *et al.* (2009 ▸); Kataoka *et al.* (2015 ▸); Gray & Walters-Smith (2011 ▸). For the synthesis, polymorphism, stability and bioavailabitily of febuxostat, see: Hiramatsu *et al.* (2000 ▸); Maddileti *et al.* (2013 ▸). For the crystal structures of febuxostat pyridine solvate and febuxostat methanol solvate, see: Zhu *et al.* (2009 ▸); Jiang *et al.* (2011 ▸).
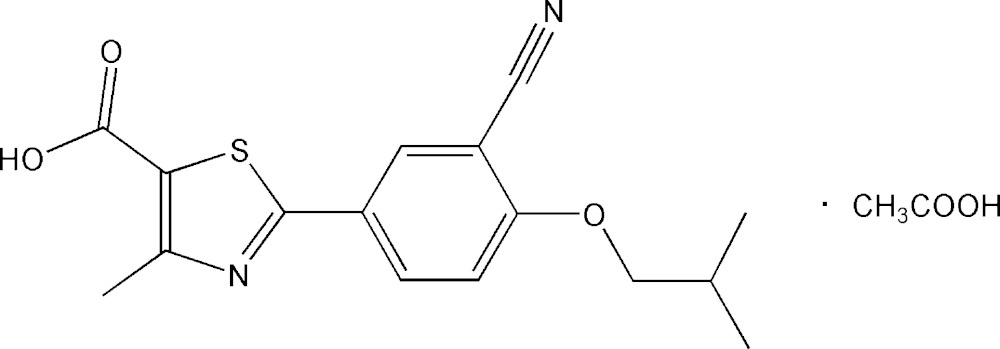


## Experimental   

### Crystal data  

C_16_H_16_N_2_O_3_S·C_2_H_4_O_2_
*M* *_r_* = 376.42
Triclinic, 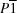

*a* = 7.684 (2) Å
*b* = 10.580 (3) Å
*c* = 12.059 (3) Å
α = 84.897 (5)°
β = 84.674 (4)°
γ = 71.081 (5)°
*V* = 921.6 (4) Å^3^
*Z* = 2
Mo *K*α radiation
μ = 0.21 mm^−1^
*T* = 296 K
0.51 × 0.30 × 0.24 mm

### Data collection  

Rigaku R-AXIS RAPID/ZJUG diffractometer
Absorption correction: multi-scan (*ABSCOR*; Higashi, 1995 ▸) *T*_min_ = 0.890, *T*_max_ = 0.952
7415 measured reflections
3397 independent reflections
2749 reflections with *I* > 2σ(*I*)
*R*_int_ = 0.026

### Refinement  

*R*[*F* ^2^ > 2σ(*F* ^2^)] = 0.040
*wR*(*F* ^2^) = 0.123
*S* = 1.00
3397 reflections
241 parameters
H-atom parameters constrained
Δρ_max_ = 0.25 e Å^−3^
Δρ_min_ = −0.26 e Å^−3^

### 

Data collection: *PROCESS-AUTO* (Rigaku, 2006 ▸); cell refinement: *PROCESS-AUTO*; data reduction: *CrystalStructure* (Rigaku, 2007 ▸); program(s) used to solve structure: *SHELXS97* (Sheldrick, 2008 ▸); program(s) used to refine structure: *SHELXL97* (Sheldrick, 2008 ▸); molecular graphics: *ORTEP-3 for Windows* (Farrugia, 2012 ▸); software used to prepare material for publication: *WinGX* (Farrugia, 2012 ▸).

## Supplementary Material

Crystal structure: contains datablock(s) I, global. DOI: 10.1107/S2056989015005708/xu5836sup1.cif

Structure factors: contains datablock(s) I. DOI: 10.1107/S2056989015005708/xu5836Isup2.hkl

Supporting information file. DOI: 10.1107/S2056989015005708/xu5836Isup3.cml

**Fig. 1.**
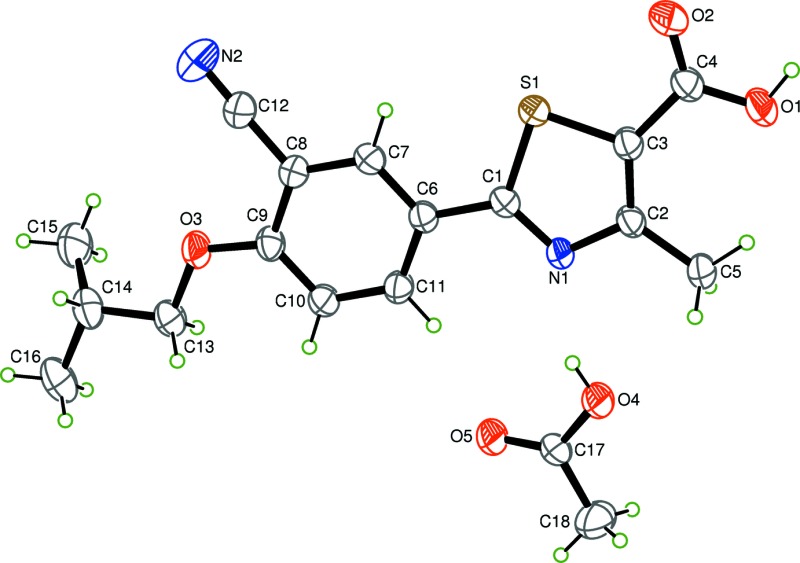
Molecular structure of the title compound (I) showing atom-labelling scheme.

. DOI: 10.1107/S2056989015005708/xu5836fig1.tif
Mol­ecular structure of the title compound (I) showing atom-labelling scheme.

**Fig. 2.**
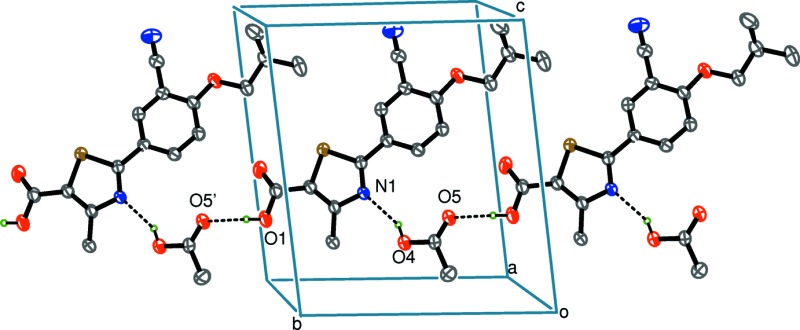
Part of the crystal packing of the title compound. Hydrogen bonds are shown as dashed lines. H atoms not involved in hydrogen bonding have been omitted for clarity.

. DOI: 10.1107/S2056989015005708/xu5836fig2.tif
Part of the crystal packing of the title compound. Hydrogen bonds are shown as dashed lines. H atoms not involved in hydrogen bonding have been omitted for clarity.

CCDC reference: 1055245

Additional supporting information:  crystallographic information; 3D view; checkCIF report

## Figures and Tables

**Table 1:** Hydrogen-bond geometry (, )

*D*H*A*	*D*H	H*A*	*D* *A*	*D*H*A*
O1H1O5^i^	0.82	1.87	2.691(2)	177
O4H4N1	0.82	2.05	2.800(3)	152
C10H10O2^ii^	0.93	2.30	3.192(3)	162
C11H11O5	0.93	2.45	3.344(3)	161

Symmetry codes: (i) 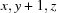; (ii) 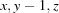.

## supplementary crystallographic information

### S1. Comment

Febuxostat, an inhibitor of xanthine oxidase, was granted marketing authorization by the European Commission for the treatment of chronic hyperuricaemia in May 2008 (Pascual *et al.*, 2009). Gout is one of the oldest *meta*-bolic diseases described, frequently categorized as a type of inflammatory arthritis, however, febuxostat is efficacious as a second-line therapy in lowering serum uric acid levels in patients with gout (Gray & Walters-Smith, 2011). According to a recent report, febuxostat has the potential usefulness for reducing cell death-induced inflammation (Kataoka *et al.*, 2015). For the important role of febuxotat, many papers and patents have been reported on the synthesis, polymorphism, stability and bioavailabitily of this drug (Hiramatsu *et al.*, 2000). For the crystal structure of febuxostat form Q, febuxostat pyridine solvate and methanol solvate has been reported. In the present study, we report the crystal structure of febuxostat acetic acid solvate. The asymmetric unit consists of one febuxostat molecule and one acetic molecule (Fig. 1), which is linked by intramolecular hydrogen bond O4—H4···N1. The benzene and thiazole rings of the febuxostat molecule are alomost coplanar with the dihedral angle of 3.24 (2)°, which is comparable with that of found (Jiang *et al.*, 2011). The carbonyl group is twist slightly to the connected benzene ring plane, as indicated by torsion angles O2—C3—C4—S1 and O1—C3—C4—C2 of 7.2 (3)° and 10.4 (4)°, respectively, which is different to that of febuxostat methanol solvate and febuxostat pyridine solvate. The cyano group is not coplanar to the benzene ring as indicated by torsion angle C7—C8—C12—N2 and C9—C8—C12—N2. Conformation of the febuxostat molecule in the structure of title compound is different silghtly to that of febuxostat methanol solvate and febuxostat pyridine solvate. In the crystal structure, acetic acid molecule is linked to the febuxostat *via* intermolecular hydrogen bond O1—H1···O5 ^i^ [symmetric code: (i)*x*,*y* + 1,*z*] hydrogen bond and intramolecular hydrogen bond O4—H4···N1 (Table 1). In this way, an infinite molecule chain is formed stretching along the the *b* axis.

### S2. Experimental

The crude product supplied by Zhejiang Huadong Pharmaceutical Co., Ltd, was recrystallized from the acetic acid solution giving colourless crystals suitable for X-ray diffraction.

### S3. Refinement

All H atoms were placed in calculated positions with O—H = 0.82 Å and C—H = 0.93–0.98 Å and included in the refinement in riding model, with *U*_iso_(H)= 1.2*U*_eq_ or 1.5*U*_eq_(carrier atom).

### Crystal data

**Table:**

C_16_H_16_N_2_O_3_S·C_2_H_4_O_2_	*Z* = 2
*M**_r_* = 376.42	*F*(000) = 396
Triclinic, *P*1	*D*_x_ = 1.356 Mg m^−^^3^
Hall symbol: -P 1	Mo *K*α radiation, λ = 0.71073 Å
*a* = 7.684 (2) Å	Cell parameters from 2789 reflections
*b* = 10.580 (3) Å	θ = 3.2–27.4°
*c* = 12.059 (3) Å	µ = 0.21 mm^−^^1^
α = 84.897 (5)°	*T* = 296 K
β = 84.674 (4)°	Chunk, colorless
γ = 71.081 (5)°	0.51 × 0.30 × 0.24 mm
*V* = 921.6 (4) Å^3^	

### Data collection

**Table:**

Rigaku R-AXIS RAPID/ZJUG diffractometer	3397 independent reflections
Radiation source: rolling anode	2749 reflections with *I* > 2σ(*I*)
Graphite monochromator	*R*_int_ = 0.026
Detector resolution: 10.00 pixels mm^-1^	θ_max_ = 25.5°, θ_min_ = 3.2°
ω scans	*h* = −8→9
Absorption correction: multi-scan (*ABSCOR*; Higashi, 1995)	*k* = −12→12
*T*_min_ = 0.890, *T*_max_ = 0.952	*l* = −14→14
7415 measured reflections	

### Refinement

**Table:**

Refinement on *F*^2^	Primary atom site location: structure-invariant direct methods
Least-squares matrix: full	Secondary atom site location: difference Fourier map
*R*[*F*^2^ > 2σ(*F*^2^)] = 0.040	Hydrogen site location: inferred from neighbouring sites
*wR*(*F*^2^) = 0.123	H-atom parameters constrained
*S* = 1.00	*w* = 1/[σ^2^(*F*_o_^2^) + (0.0641*P*)^2^ + 0.4268*P*] where *P* = (*F*_o_^2^ + 2*F*_c_^2^)/3
3397 reflections	(Δ/σ)_max_ < 0.001
241 parameters	Δρ_max_ = 0.25 e Å^−^^3^
0 restraints	Δρ_min_ = −0.26 e Å^−^^3^

### Special details

**Table:**

Geometry. All e.s.d.'s (except the e.s.d. in the dihedral angle between two l.s. planes) are estimated using the full covariance matrix. The cell e.s.d.'s are taken into account individually in the estimation of e.s.d.'s in distances, angles and torsion angles; correlations between e.s.d.'s in cell parameters are only used when they are defined by crystal symmetry. An approximate (isotropic) treatment of cell e.s.d.'s is used for estimating e.s.d.'s involving l.s. planes.
Refinement. Refinement of *F*^2^ against ALL reflections. The weighted *R*-factor *wR* and goodness of fit *S* are based on *F*^2^, conventional *R*-factors *R* are based on *F*, with *F* set to zero for negative *F*^2^. The threshold expression of *F*^2^ > σ(*F*^2^) is used only for calculating *R*-factors(gt) *etc*. and is not relevant to the choice of reflections for refinement. *R*-factors based on *F*^2^ are statistically about twice as large as those based on *F*, and *R*- factors based on ALL data will be even larger.

### Fractional atomic coordinates and isotropic or equivalent isotropic displacement parameters (Å^2^)

**Table:**

	*x*	*y*	*z*	*U*_iso_*/*U*_eq_	
S1	0.72628 (8)	0.73569 (5)	0.50085 (4)	0.03662 (17)	
O1	0.5945 (3)	1.02293 (15)	0.26948 (14)	0.0507 (4)	
H1	0.5889	1.1016	0.2705	0.076*	
O2	0.7074 (3)	1.00765 (16)	0.43526 (15)	0.0584 (5)	
O3	0.8936 (2)	0.12754 (14)	0.74240 (13)	0.0435 (4)	
O4	0.5922 (3)	0.46989 (15)	0.17802 (14)	0.0538 (5)	
H4	0.6221	0.4821	0.2387	0.081*	
O5	0.5837 (3)	0.28003 (15)	0.26573 (13)	0.0511 (4)	
N1	0.6762 (2)	0.60007 (15)	0.34796 (13)	0.0305 (4)	
N2	0.9313 (4)	0.3647 (3)	0.90533 (19)	0.0707 (7)	
C3	0.6693 (3)	0.81547 (19)	0.37300 (17)	0.0324 (5)	
C2	0.6483 (3)	0.72860 (18)	0.30167 (17)	0.0304 (4)	
C1	0.7188 (3)	0.59016 (18)	0.45268 (16)	0.0291 (4)	
C4	0.6599 (3)	0.9576 (2)	0.36255 (18)	0.0364 (5)	
C5	0.6022 (3)	0.7595 (2)	0.18262 (18)	0.0416 (5)	
H5A	0.4835	0.7508	0.1747	0.062*	
H5B	0.6938	0.6981	0.1365	0.062*	
H5C	0.5997	0.8493	0.1601	0.062*	
C6	0.7591 (3)	0.46731 (19)	0.52647 (16)	0.0299 (4)	
C11	0.7480 (3)	0.3481 (2)	0.49263 (17)	0.0333 (5)	
H11	0.7106	0.3464	0.4218	0.040*	
C10	0.7910 (3)	0.2323 (2)	0.56132 (18)	0.0361 (5)	
H10	0.7836	0.1537	0.5363	0.043*	
C9	0.8457 (3)	0.2333 (2)	0.66802 (17)	0.0332 (5)	
C8	0.8528 (3)	0.3538 (2)	0.70422 (17)	0.0332 (5)	
C7	0.8122 (3)	0.4681 (2)	0.63359 (17)	0.0337 (5)	
H7	0.8205	0.5468	0.6580	0.040*	
C13	0.8752 (3)	0.0017 (2)	0.71520 (19)	0.0412 (5)	
H13A	0.9542	−0.0312	0.6495	0.049*	
H13B	0.7487	0.0139	0.7003	0.049*	
C14	0.9314 (3)	−0.0965 (2)	0.8147 (2)	0.0450 (6)	
H14	1.0592	−0.1062	0.8277	0.054*	
C15	0.8132 (4)	−0.0512 (3)	0.9196 (2)	0.0650 (8)	
H15A	0.8197	0.0342	0.9362	0.098*	
H15B	0.8569	−0.1154	0.9804	0.098*	
H15C	0.6878	−0.0434	0.9091	0.098*	
C16	0.9239 (4)	−0.2326 (2)	0.7854 (3)	0.0605 (7)	
H16A	0.8005	−0.2242	0.7692	0.091*	
H16B	0.9601	−0.2966	0.8474	0.091*	
H16C	1.0064	−0.2622	0.7212	0.091*	
C12	0.8996 (3)	0.3588 (2)	0.81619 (19)	0.0440 (5)	
C17	0.5719 (3)	0.3514 (2)	0.18152 (18)	0.0371 (5)	
C18	0.5320 (5)	0.3165 (3)	0.0724 (2)	0.0676 (8)	
H18A	0.5375	0.2243	0.0769	0.101*	
H18B	0.6217	0.3305	0.0159	0.101*	
H18C	0.4111	0.3722	0.0538	0.101*	

### Atomic displacement parameters (Å^2^)

**Table:**

	*U*^11^	*U*^22^	*U*^33^	*U*^12^	*U*^13^	*U*^23^
S1	0.0516 (3)	0.0284 (3)	0.0330 (3)	−0.0157 (2)	−0.0089 (2)	−0.0002 (2)
O1	0.0769 (12)	0.0284 (8)	0.0533 (10)	−0.0232 (8)	−0.0232 (9)	0.0079 (7)
O2	0.0934 (14)	0.0360 (9)	0.0574 (11)	−0.0312 (9)	−0.0266 (10)	−0.0004 (8)
O3	0.0615 (10)	0.0317 (8)	0.0401 (9)	−0.0190 (7)	−0.0145 (7)	0.0104 (6)
O4	0.0965 (14)	0.0329 (8)	0.0406 (9)	−0.0315 (9)	−0.0159 (9)	0.0059 (7)
O5	0.0845 (13)	0.0324 (8)	0.0425 (9)	−0.0267 (8)	−0.0148 (8)	0.0068 (7)
N1	0.0390 (9)	0.0238 (8)	0.0294 (9)	−0.0111 (7)	−0.0063 (7)	0.0025 (6)
N2	0.0975 (19)	0.0752 (16)	0.0407 (13)	−0.0255 (15)	−0.0214 (12)	−0.0011 (11)
C3	0.0372 (11)	0.0281 (10)	0.0332 (11)	−0.0122 (8)	−0.0059 (9)	0.0028 (8)
C2	0.0341 (10)	0.0246 (9)	0.0332 (11)	−0.0110 (8)	−0.0032 (8)	0.0018 (8)
C1	0.0295 (10)	0.0254 (9)	0.0320 (10)	−0.0089 (8)	−0.0008 (8)	−0.0012 (8)
C4	0.0422 (12)	0.0291 (10)	0.0406 (12)	−0.0153 (9)	−0.0052 (9)	0.0013 (9)
C5	0.0627 (14)	0.0295 (10)	0.0352 (12)	−0.0177 (10)	−0.0129 (10)	0.0063 (9)
C6	0.0314 (10)	0.0274 (10)	0.0302 (10)	−0.0090 (8)	−0.0017 (8)	0.0008 (8)
C11	0.0388 (11)	0.0327 (10)	0.0304 (10)	−0.0141 (9)	−0.0061 (9)	0.0023 (8)
C10	0.0462 (12)	0.0292 (10)	0.0364 (11)	−0.0161 (9)	−0.0074 (9)	0.0009 (9)
C9	0.0367 (11)	0.0303 (10)	0.0337 (11)	−0.0136 (9)	−0.0042 (9)	0.0057 (8)
C8	0.0375 (11)	0.0329 (10)	0.0286 (10)	−0.0104 (9)	−0.0039 (8)	0.0009 (8)
C7	0.0404 (11)	0.0279 (10)	0.0338 (11)	−0.0120 (9)	−0.0031 (9)	−0.0017 (8)
C13	0.0506 (13)	0.0313 (11)	0.0437 (13)	−0.0173 (10)	−0.0039 (10)	0.0049 (9)
C14	0.0450 (12)	0.0355 (11)	0.0528 (14)	−0.0126 (10)	−0.0094 (11)	0.0110 (10)
C15	0.088 (2)	0.0519 (15)	0.0481 (15)	−0.0164 (15)	−0.0039 (14)	0.0141 (12)
C16	0.0697 (17)	0.0333 (12)	0.0743 (19)	−0.0146 (12)	−0.0043 (15)	0.0111 (12)
C12	0.0577 (14)	0.0389 (12)	0.0357 (13)	−0.0157 (11)	−0.0094 (10)	0.0032 (9)
C17	0.0473 (12)	0.0272 (10)	0.0388 (12)	−0.0148 (9)	−0.0030 (9)	−0.0010 (9)
C18	0.119 (3)	0.0564 (16)	0.0429 (15)	−0.0473 (17)	−0.0126 (15)	−0.0017 (12)

### Geometric parameters (Å, º)

**Table:**

S1—C3	1.713 (2)	C11—H11	0.9300
S1—C1	1.713 (2)	C10—C9	1.392 (3)
O1—C4	1.316 (3)	C10—H10	0.9300
O1—H1	0.8200	C9—C8	1.403 (3)
O2—C4	1.204 (3)	C8—C7	1.381 (3)
O3—C9	1.344 (2)	C8—C12	1.439 (3)
O3—C13	1.452 (3)	C7—H7	0.9300
O4—C17	1.309 (3)	C13—C14	1.512 (3)
O4—H4	0.8200	C13—H13A	0.9700
O5—C17	1.203 (3)	C13—H13B	0.9700
N1—C1	1.321 (3)	C14—C15	1.506 (4)
N1—C2	1.380 (2)	C14—C16	1.533 (3)
N2—C12	1.135 (3)	C14—H14	0.9800
C3—C2	1.369 (3)	C15—H15A	0.9600
C3—C4	1.477 (3)	C15—H15B	0.9600
C2—C5	1.493 (3)	C15—H15C	0.9600
C1—C6	1.470 (3)	C16—H16A	0.9600
C5—H5A	0.9600	C16—H16B	0.9600
C5—H5B	0.9600	C16—H16C	0.9600
C5—H5C	0.9600	C17—C18	1.482 (3)
C6—C11	1.389 (3)	C18—H18A	0.9600
C6—C7	1.392 (3)	C18—H18B	0.9600
C11—C10	1.380 (3)	C18—H18C	0.9600
			
C3—S1—C1	89.55 (10)	C8—C7—C6	120.94 (19)
C4—O1—H1	109.5	C8—C7—H7	119.5
C9—O3—C13	118.74 (17)	C6—C7—H7	119.5
C17—O4—H4	109.5	O3—C13—C14	107.20 (18)
C1—N1—C2	110.89 (16)	O3—C13—H13A	110.3
C2—C3—C4	134.5 (2)	C14—C13—H13A	110.3
C2—C3—S1	110.65 (15)	O3—C13—H13B	110.3
C4—C3—S1	114.82 (15)	C14—C13—H13B	110.3
C3—C2—N1	114.23 (18)	H13A—C13—H13B	108.5
C3—C2—C5	126.75 (18)	C15—C14—C13	112.9 (2)
N1—C2—C5	119.01 (17)	C15—C14—C16	110.9 (2)
N1—C1—C6	125.29 (17)	C13—C14—C16	108.2 (2)
N1—C1—S1	114.68 (14)	C15—C14—H14	108.2
C6—C1—S1	120.03 (15)	C13—C14—H14	108.2
O2—C4—O1	123.93 (19)	C16—C14—H14	108.2
O2—C4—C3	121.4 (2)	C14—C15—H15A	109.5
O1—C4—C3	114.69 (18)	C14—C15—H15B	109.5
C2—C5—H5A	109.5	H15A—C15—H15B	109.5
C2—C5—H5B	109.5	C14—C15—H15C	109.5
H5A—C5—H5B	109.5	H15A—C15—H15C	109.5
C2—C5—H5C	109.5	H15B—C15—H15C	109.5
H5A—C5—H5C	109.5	C14—C16—H16A	109.5
H5B—C5—H5C	109.5	C14—C16—H16B	109.5
C11—C6—C7	118.10 (18)	H16A—C16—H16B	109.5
C11—C6—C1	122.16 (18)	C14—C16—H16C	109.5
C7—C6—C1	119.74 (18)	H16A—C16—H16C	109.5
C10—C11—C6	121.79 (19)	H16B—C16—H16C	109.5
C10—C11—H11	119.1	N2—C12—C8	178.0 (3)
C6—C11—H11	119.1	O5—C17—O4	122.5 (2)
C11—C10—C9	119.94 (19)	O5—C17—C18	124.4 (2)
C11—C10—H10	120.0	O4—C17—C18	113.11 (19)
C9—C10—H10	120.0	C17—C18—H18A	109.5
O3—C9—C10	125.95 (18)	C17—C18—H18B	109.5
O3—C9—C8	115.24 (18)	H18A—C18—H18B	109.5
C10—C9—C8	118.81 (18)	C17—C18—H18C	109.5
C7—C8—C9	120.38 (19)	H18A—C18—H18C	109.5
C7—C8—C12	119.81 (19)	H18B—C18—H18C	109.5
C9—C8—C12	119.80 (18)		
			
C1—S1—C3—C2	−0.09 (16)	S1—C1—C6—C7	−3.2 (3)
C1—S1—C3—C4	−178.19 (16)	C7—C6—C11—C10	−1.1 (3)
C4—C3—C2—N1	177.9 (2)	C1—C6—C11—C10	178.15 (18)
S1—C3—C2—N1	0.3 (2)	C6—C11—C10—C9	0.6 (3)
C4—C3—C2—C5	−1.3 (4)	C13—O3—C9—C10	−5.0 (3)
S1—C3—C2—C5	−178.87 (18)	C13—O3—C9—C8	174.90 (18)
C1—N1—C2—C3	−0.4 (2)	C11—C10—C9—O3	−179.0 (2)
C1—N1—C2—C5	178.84 (18)	C11—C10—C9—C8	1.1 (3)
C2—N1—C1—C6	−179.40 (18)	O3—C9—C8—C7	177.91 (18)
C2—N1—C1—S1	0.3 (2)	C10—C9—C8—C7	−2.2 (3)
C3—S1—C1—N1	−0.13 (16)	O3—C9—C8—C12	−3.1 (3)
C3—S1—C1—C6	179.60 (16)	C10—C9—C8—C12	176.8 (2)
C2—C3—C4—O2	−170.3 (2)	C9—C8—C7—C6	1.6 (3)
S1—C3—C4—O2	7.2 (3)	C12—C8—C7—C6	−177.3 (2)
C2—C3—C4—O1	10.4 (4)	C11—C6—C7—C8	0.0 (3)
S1—C3—C4—O1	−172.07 (16)	C1—C6—C7—C8	−179.29 (18)
N1—C1—C6—C11	−2.8 (3)	C9—O3—C13—C14	−177.85 (18)
S1—C1—C6—C11	177.53 (15)	O3—C13—C14—C15	60.1 (3)
N1—C1—C6—C7	176.48 (19)	O3—C13—C14—C16	−176.75 (19)

### Hydrogen-bond geometry (Å, º)

**Table:**

*D*—H···*A*	*D*—H	H···*A*	*D*···*A*	*D*—H···*A*
O1—H1···O5^i^	0.82	1.87	2.691 (2)	177
O4—H4···N1	0.82	2.05	2.800 (3)	152
C10—H10···O2^ii^	0.93	2.30	3.192 (3)	162
C11—H11···O5	0.93	2.45	3.344 (3)	161

Symmetry codes: (i) *x*, *y*+1, *z*; (ii) *x*, *y*−1, *z*.
