# Supplementary figures and images for: Crystal structure of febuxostat–acetic acid (1/1)
Source: Acta Crystallogr E Crystallogr Commun. 2015 Apr 9;71(Pt 5):o295–6. doi: 10.1107/S2056989015005708 (PMC4420075; doi:10.1107/S2056989015005708)

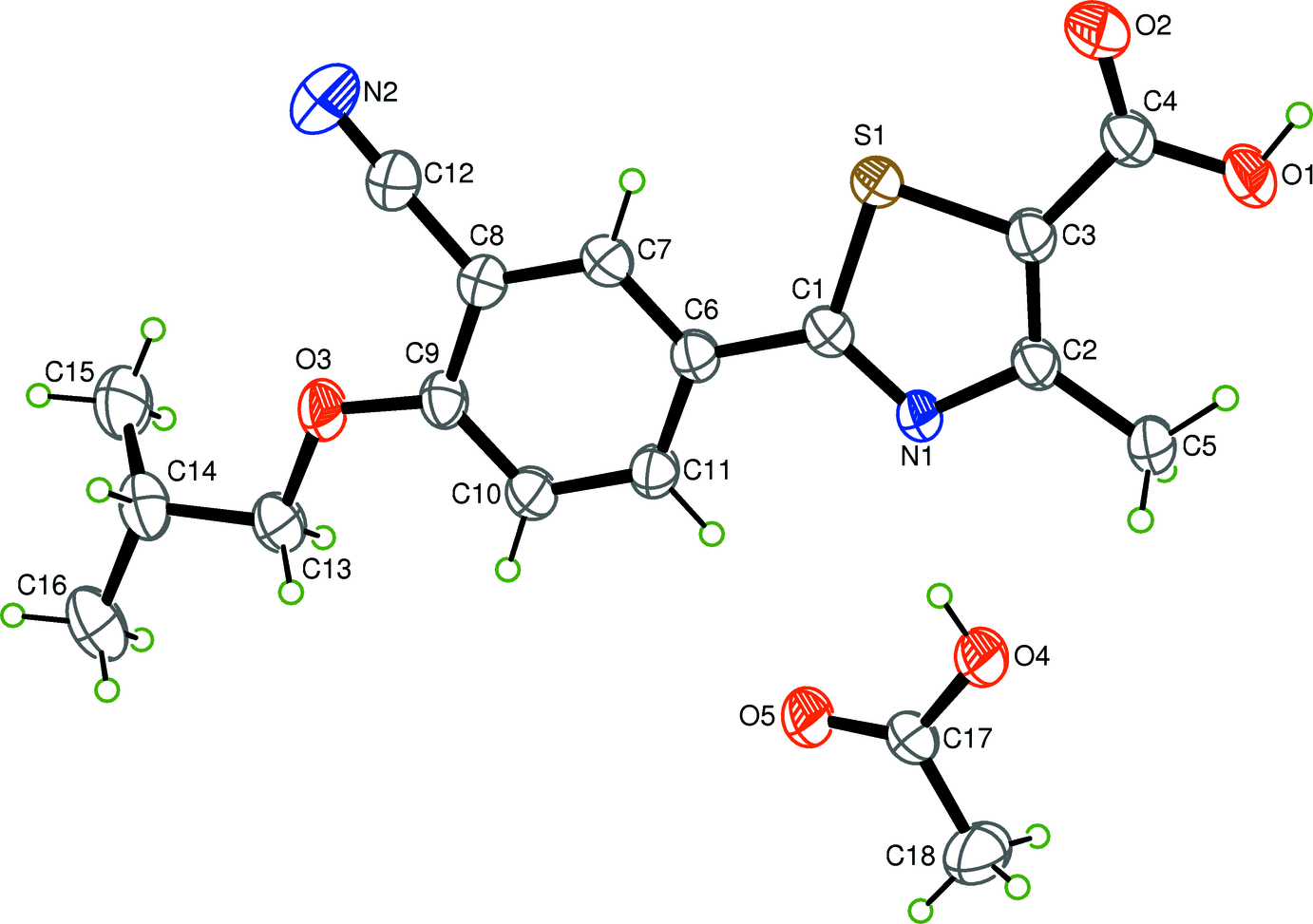

Supplement: Supplementary file 4 [file e-71-0o295-fig1.tif]

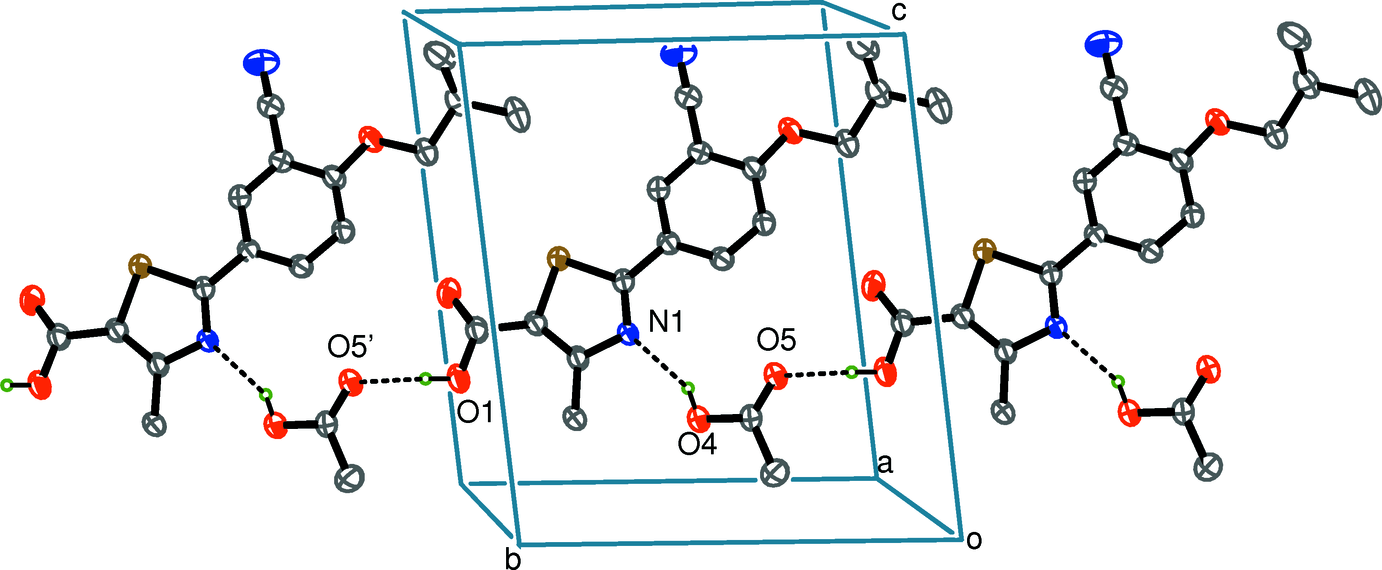

Supplement: Supplementary file 5 [file e-71-0o295-fig2.tif]
